# Factors affecting the development of airflow limitation among South Korean smokers

**DOI:** 10.18332/tid/103088

**Published:** 2019-03-01

**Authors:** Youngmee Kim, Won-Kyung Cho

**Affiliations:** 1 Red Cross College of Nursing, Chung-Ang University, Seoul, Korea; 2 Department of Pulmonary and Critical Care Medicine, International Healthcare Center, Asan Medical Center, University of Ulsan College of Medicine, Seoul, Korea

**Keywords:** smokers, airflow limitation, Koreans

## Abstract

**INTRODUCTION:**

Considering that only some and not all smokers develop chronic obstructive pulmonary disease (COPD), there may be other factors responsible for the development of COPD among smokers. Previous researchers have reported that ethnicity could be one of factors associated with the development of COPD. This study aimed to examine the factors associated with the development of airflow limitation, suggestive of having COPD, among Korean smokers using data from the Korea National Health and Nutrition Examination Survey conducted over the past 3 years.

**METHODS:**

A total of 2569 current and former smokers ≥40 years of age were included. Most studies exploring risk factors for COPD have compared smokers and non-smokers with smoking as only one of the independent variables. In this study, we took a different approach, studying only smokers and comparing those with or without airflow limitation.

**RESULTS:**

The prevalence of airflow limitation among the study participants was 19.2% and 22.1% in current and former smokers, respectively. There was no significant correlation between the severity of airflow limitation and total lifetime smoking amount. Among the many variables examined, only age, male gender and total lifetime smoking amount (pack-years) were significant factors associated with the development of cigarette smoke-induced airflow limitation.

**CONSLUSIONS:**

Older Korean men who are heavy smokers may be at a higher risk of developing COPD. Our findings support the importance of smoking cessation as the best way to prevent the development of COPD.

## INTRODUCTION

Chronic obstructive pulmonary disease (COPD) is one of the leading causes of death worldwide. As of 2015, COPD affected about 174.5 million (2.4%) of the global population^1^. Approximately three million people died of COPD in 2015, accounting for 5% of all deaths worldwide in that year^2^. By 2030, COPD is expected to rank 4th on the list of causes of death and 7th in terms of disease burden^7^. In Korea, the prevalence of cigarette smoking and COPD among adults in 2016 was 23.9% and 12.1%, respectively. Due to its high prevalence, mortality and chronicity, COPD has been a global burden for many years. The Global Initiative for Chronic Obstructive Lung Disease (GOLD), led by the National Heart, Lung, and Blood Institute (NHLBI) and the World Health Organization (WHO), defines COPD as ‘a common, preventable and treatable disease that is characterised by persistent respiratory symptoms and airflow limitation that is due to airway and/or alveolar abnormalities usually caused by significant exposure to noxious particles or gases’^3^. Although environmental exposures such as air pollution, secondhand smoke and biomass smoke (e.g. animal dung and crop residues) and genetic factors may contribute to the development of COPD, smoking is predominantly the major cause^4-6^. The risk attributable to active smoking in COPD has been reported to be 40–70%^7^. Previously, it was considered that only 15–20% of smokers develop clinically significant COPD^8^; however, recent studies have reported that about 50% of smokers eventually develop COPD during their lifetime^9,10^. Given that only some smokers develop COPD, it is important to identify the factors that may influence the development of COPD among smokers. It has been reported that various factors, such as aging^11,12^, gender^13,14^, abnormal lung growth and development^5^, occupational exposure to particles^15^, air pollution^16^, infection^17^, lower socioeconomic status^18-20^, nutrition^21^, obesity^22^ and educational level^23^, were associated with a higher risk of developing cigarette smoke-induced COPD. Previous researchers have also reported that COPD risk varies according to ethnicity^24,25^. Therefore, some studies have examined the factors associated with COPD according to ethnicity or country; however, the risk factors identified were predominantly the same as those stated above^17,18,26-29^.

The objectives of the present study were to examine the following: 1) the prevalence of airflow limitation among smokers, suggestive of COPD; 2) the factors associated with airflow limitation among smokers, and 3) the relationship between smoking history and severity of airflow limitation. To address these, we analysed the data from the Korea National Health and Nutrition Examination Survey (KNHANES). The KNHANES is an ongoing, nationally representative, annual cross-sectional survey compiling health data of the civilian population in South Korea^30^.

## METHODS

### Data source and collection

**Figure 1:**
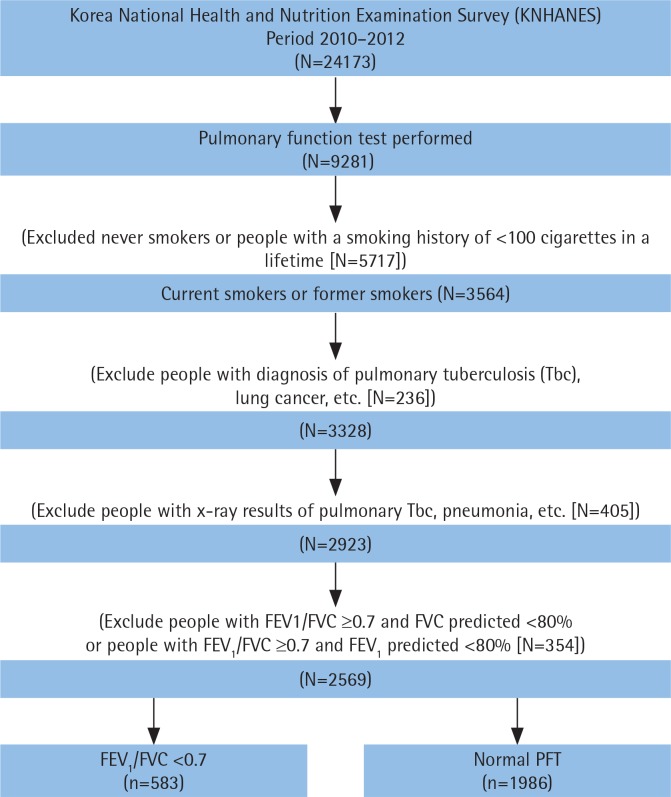
Flow diagram showing inclusion and exclusion of studies

The present study was a secondary data analysis of KNHANES data from 2010 to 2012 (the fifth survey) conducted by the Korea Centers for Disease Control and Prevention (KCDC). Since the inclusion and exclusion criteria for this study required chest x-ray data, as described below, only the fifth survey data that included chest x-ray information were used. The KNHANES consists of a health interview survey, a health examination survey and a nutritional survey. To minimise sampling bias, the KNHANES used a complex, stratified multistage probability cluster sampling design based on regional areas, sex and age. Further information about the study design and sampling process are provided elsewhere^30^. A total of 24173 individuals participated in the fifth (2010–2012) KNHANES survey. From among them, individuals who had undergone spirometry and smoked at least 100 cigarettes in their lifetime, regardless of current smoking status^31,32^, were included in the analysis. Records of individuals were excluded from the study if they: had never smoked or had smoked fewer than 100 cigarettes in their lifetime; had abnormal CXR findings due to lung diseases other than COPD as confirmed by a radiologist; had a history of other pulmonary diseases such as asthma, sarcoidosis, pulmonary tuberculosis, lung cancer or interstitial lung disease; and those who had a forced expiratory volume in 1 s (FEV_1_)/forced vital capacity (FVC) ratio ≥0.7 with < 80% of FVC to exclude the participants with restrictive lung disease^33^. Because spirometry was performed in individuals ≥40 years of age in the survey, individuals ≥40 years of age were included in this study. Therefore, a total of 2569 participants were included in this study. Figure 1 demonstrates the selection process and the number of study participants.

### Measures

#### Spirometry

Spirometry was performed according to the acceptability and repeatability criteria published by the American Thoracic Society. The largest FVC and FEV_1_ were used for analysis after completing at least three acceptable adjustments^34^. The difference in FVC between the largest and next largest adjustments should be within 5% or 150 mL of each other, whichever is greater. If not, additional trials were made to meet the repeatability criteria^34^. The predicted value for each individual was determined using the reference equations derived from the Korean population^35^. Airflow limitation is defined by FEV_1_/FVC ratio <0.70, according to the GOLD guidelines^33^. Airflow limitation that is only partially reversible with a bronchodilator or is completely irreversible is the characteristic physiological feature of COPD^33,36^. Therefore, both pre- and post-bronchodilator spirometry data are needed to diagnose COPD. However, only pre-bronchodilator spirometry data were available for this study. The severity of airflow limitation is determined by the per cent predicted value of FEV_1_, thus the ranges FEV_1_≥80%, 50%≤FEV_1_<80%, 30%≤FEV_1_<50% and 30%<FEV_1_ are observed in patients with mild, moderate, severe, and very severe airflow limitation, respectively^33,34^.

#### Clinical diseases

Diabetes mellitus (DM) was defined as a fasting blood glucose level ≥126 mg/dL, taking oral hypoglycaemic agents or insulin injection or having been diagnosed with DM. Hypertension was defined as a systolic blood pressure ≥140 mmHg, a diastolic blood pressure ≥90 mmHg or taking antihypertensive medications. Other diseases were defined based on a participant’s medical history^30^.

#### Health behaviours, perceived health status and mental health

The definition of smokers in our study was based on the guidelines of the Centers for Disease Control and Prevention (CDC): non-smokers were those who smoked <100 cigarettes in their lifetime, current smokers were those who smoked ≥100 cigarettes in their lifetime and were current smokers at the time of the survey, and ex-smokers were those who smoked >100 cigarettes in their lifetime but quit smoking at the time of the survey^31,32^. Lifetime smoking amount (pack-years) was calculated by multiplying the number of packs of cigarettes smoked per day by the number of years the person smoked. Alcohol drinking was defined as the consumption of more than seven glasses of alcoholic drink per occasion, more than two times per week. Regular exercise was defined as a moderate level of physical exercise for ≥30 min at a time ≥5 times per week^30^. Perceived health status was categorised into different groups — very good/good, fair or poor/very poor by self-rated levels of health status. Health-related quality of life (HRQoL) was evaluated using the EuroQoL^37^. The EuroQoL measures both a health-status descriptive system (EQ-5D) and a visual analogue scale (EQVAS), and ranges from 0 to 100, where zero signifies worst health condition and 100 designates best health condition^37^. Stress was defined as stress perceived to be moderate to severe. Depressive symptoms were defined as feelings of sadness disruptive to one’s activities of daily living for at least two consecutive weeks in the past year. Suicidal ideation was defined as intention of committing suicide during the past year^30^.

### Statistical analysis

All data were analysed using SAS version 9.3 (SAS Institute Inc., NC, USA); p<0.05 was considered to be statistically significant. The data are presented as mean ± standard error (SE) for continuous variables or proportions for categorical variables. T-tests, ANOVA and χ^2^ tests were performed to evaluate the differences among the groups for continuous and categorical variables, respectively. Multiple logistic regression analyses were performed to explore the associations between airflow limitation and various factors. Variables that were statistically significant in the univariate tests were selected for the multivariate analyses. The results were reported using adjusted odd ratios (ORs) and their 95% confidential interval (CI). A 95% CI that did not span 1.0 was considered to be statistically significant.

## RESULTS

### Prevalence of airflow limitation and mean ages of the study participants

Table 1 summarise the prevalence of airflow limitation and mean ages of the study participants according to their smoking status. The prevalence of airflow limitation among the study participants was 19.2% and 22.1% in current and former smokers, respectively. The mean ages of the study participants were 51.40 and 55.97 years among current and former smokers, respectively. The mean age of study participants with airflow limitation was 60.98±0.78 and 64.83±0.82 years among current and former smokers, respectively. Regardless of smoking history, participants with airflow limitation were older than those with normal spirometry results. In addition, Supplementary Tables 1.1 and 1.2 summarise the prevalence of airflow limitation and mean ages of the entire population for whom spirometry was performed, including non-smokers.

**Table 1:** Prevalence of airflow limitation and mean age (years) among the study participants (current and former smokers)

*Variables*	*Current smokers (n=1217)*		*Former smokers (n=1352)*		*p*
	*Prevalence of airflow limitation*				
	*n*	*% (SE)*	*n*	*% (SE)*	
FEV_1_/FVC<0.7	261	19.2 (1.34)	322	22.1 (1.41)	0.114
Normal spirometry	956	80.8 (1.43)	1030	77.9 (1.41)	
	***Mean age***				***p***
	***n***	***Mean±SE***	***n***	***Mean±SE***	
FEV_1_/FVC<0.7	261	60.98±0.78	322	64.83±0.82	<0.001
Normal spirometry	956	49.13±0.28	1030	53.45±0.38	<0.001
Total	1217	51.40±0.33	1352	55.97±0.40	<0.001

Prevalence of air flow limitation data are presented as weighted % (SE), p-value is by chi-squared. Mean age data are presented as weighted mean±standard error (SE), p-vales are by Student’s t-test.

### Sociodemographic characteristics and smoking history

Table 2 summarises the sociodemographic characteristics of the study participants according to the presence or absence of airflow limitation. The mean age of the group with airflow limitation was significantly higher (62.98 years) than that of the group without airflow limitation (51.17 years; p<0.001). Furthermore, the prevalence of airflow limitation increased in older populations. The prevalence of airflow limitation in participants between 40 and 55 years of age was the lowest (9.1%), and the prevalence of airflow limitation in participants ≥76 years of age was highest (65.9%; p<0.001). In addition, the prevalence of airflow limitation was significantly higher in male (p=0.024), divorced, separated or widowed (p<0.001) and unemployed (p<0.001) participants who resided in rural areas (p=0.005). Household income showed no difference between groups.

**Table 2:** Sociodemographic characteristics of study participants according to the presence or absence of airflow limitation (N = 2569)

*Variables*	*Group with airflow limitation (n=583 )*	*Group without airflow limitation (n=1986 )*	*p*
**Age (years),** mean±SE	62.98±0.57	51.17±0.25	<0.001
	***% (±SE)***	***% (±SE)***	***p***
**Age group (years)**			<0.001
40–55	9.1 (0.92)	90.9 (0.92)	
56–65	30.6 (2.35)	69.4 (2.35)	
66–75	52.2 (3.03)	47.8 (3.03)	
≥76	65.9 (5.63)	34.1 (5.63)	
**Gender**			0.024
Male	21.3 (1.08)	78.8 (1.08)	
Female	13.8 (2.67)	86.2 (2.67)	
**Marital status**			<0.001
Married	20.4 (1.05)	79.6 (1.05)	
	Never-married	5.6 (2.55)	94.4 (2.55)
Other (divorced, separated, widowed)	29.5 (3.82)	70.5 (3.82)	
**Employment types**			<0.001
Clerical work	11.5 (1.33)	88.5 (1.33)	
Labor work	20.7 (1.44)	79.3 (1.44)	
Unemployed	37.2 (2.59)	62.8 (2.59)	
**Living place**			0.005
Urban	18.9 (1.08)	81.1 (1.08)	
Rural	25.5 (2.26)	74.5 (2.26)	
**Household income** (% in quartiles)			0.506
1 Lowest	20.4 (1.91)	79.6 (1.91)	
2	23.0 (1.88)	77.0 (1.88)	
3	19.9 (2.03)	80.1 (2.03)	
4 Highest	19.1 (2.03)	80.9 (2.03)	

Data are presented as weighted mean±standard error (SE) or weighted % (±SE); T-test or chi-squared test was adopted for continuous or categorical variables, respectively. Income quartiles are age and gender adjusted.

Table 3 summarises smoking history of study participants according to the presence or absence of airflow limitation. The daily smoking amount also did not show a significant difference between two groups; however, there was a significant difference between groups in regard to years of smoking, leading to a significantly higher pack-years in the group with airflow limitation compared with the group without airflow limitation (p<0.001).

**Table 3:** Smoking history of study participants according to the presence or absence of airflow limitation (N=2569)

*Variables*	*Group with airflow limitation (n=583 )*	*Group without airflow limitation (n=1986 )*	*p*
	*% (SE)*	*% (SE)*	
**Smoker types**			0.114
Current smokers	19.2 (1.34)	80.8 (1.34)	
Former smokers	22.1 (1.41)	77.9 (1.41)	
**Current smoker**			0.342
Cigarettes per day			
<10	23.3 (3.26)	76.7 (3.26)	
10–19	18.1 (2.08)	81.9 (2.08)	
≥ 20	18.7 (1.86)	81.3 (1.86)	
Years of smoking	39.62±0.79	28.08±0.31	<0.001
Pack-years	31.80±1.37	23.84±0.58	<0.001
**Former smoker**			0.063
Cigarettes per day			
<10	19.8 (3.60)	80.2 (3.60)	
10–19	17.8 (2.36)	82.2 (2.36)	
≥ 20	25.2 (1.93)	74.8 (1.93)	
Years of smoking	26.81±1.00	18.64±0.42	<0.001
Pack-years	27.94±1.44	18.47±0.66	<0.001
Years of smoking cessation	17.37±1.01	14.55±0.37	0.009
			0.767
<1	27.3 (8.34)	72.7 (8.34)	
1 ≤ duration <10	21.2 (2.57)	78.8 (2.57)	
≥ 10	22.0 (1.84)	78.0 (1.84)	

Data are presented as weighted mean±standard error (SE) or weighted % (±SE); T-test or chi-squared test was adopted for continuous or categorical variables, respectively. Income quartiles are age and gender adjusted.

### Health behaviours, mental health, nutritional status and medical comorbidities

Table 4 summarises comorbidities, health behaviours and mental health status of study participants according to the presence or absence of airflow limitation. All of the nutritional indices measured were significantly worse in the group with airflow limitation. The group with airflow limitation had more participants with comorbidities including DM, hypertension, CVD and cancers compared with the group without airflow limitation. Based on the EuroQoL surveys, HRQoL was significantly worse in the group with airflow limitation.

**Table 4:** Comparison of health behaviors, mental health, and medical conditions between group with or without airflow limitation

*Variables*	*Group with airflow limitation*		*Group without airflow limitation*		*p*
	*n*	*Mean±SE or weighted % ±SE*	*n*	*Mean±SE or weighted % ±SE*	
SBP (mmHg)	583	126.35±0.89	1984	120.85±0.46	<0.001
DBP (mmHg)	583	77.31±0.58	1984	79.76±0.34	<0.001
BMI (kg/m^2^)	583	23.72±0.16	1086	24.42±0.07	<0.001
25(OH)D (ng/mL)	559	19.51±0.36	1944	19.24±0.22	0.467
Dietary Intake					
(Kcal/day)	529	2136.66±50.86	1686	2428.38±29.26	<0.001
Protein (g/day)	529	73.90±2.54	1686	86.09±1.25	<0.001
Fat (g/day)	529	34.23±1.47	1686	47.15±0.99	<0.001
Carbohydrate (g/day)	529	347.94±8.24	1686	369. 60±4.59	0.019
Vitamin A (ugRE)	529	854.73±60.73	1686	1008.99±39.85	0.031
Vitamin B1 (mg)	529	1.31±0.04	1686	1.61±0.03	<0.001
Vitamin B2 (mg)	529	1.19±0.04	1686	1.44±0.02	<0.001
Vitamin C (mg)	529	98.56±4.01	1686	122.58±2.63	<0.001
Total cholesterol (mg/dL)	559	187.25±2.21	1944	194.57±1.04	0.002
Diabetes mellitus (%)	544	18.57	1895	12.34	<0.001
Hypertension (%)	582	49.31	1978	35.69	<0.001
CVD (%)	583	6.00	1986	4.08	<0.001
*Cancer (%)	583	4.97	1984	2.87	0.003
Heavy drinker (%)	437	18.99	1702	28.43	0.006
Regular exerciser (%)	583	9.26	1985	10.48	0.997
Perceived health status (%)	582		1984		<0.001
Very good/good		34.53		36.39	
Fair		44.50		49.70	
Poor/very poor		20.96		13.91	
EQ-5D	582	0.92±0.01	1984	0.96±0.00	<0.001
EuroQoL:VAS	582	71.28±1.08	1982	75.32±0.45	<0.001
Perceived psychological stress (% for yes)	583	15.09	1986	24.62	<0.001
Depressive symptom (%)	583	9.78	1986	10.67	0.681
Suicide ideation (%)	583	21.0	1986	79.0	0.316

Data except comorbidity data are presented as subject number (n), weighted mean±standard error (±SE) or weighted % (±SE); T-test or chi-squared test was adopted for continuous or categorical variables, respectively.

* The patients with lung cancer were excluded among cancer patients. SBP: systolic blood pressure, DBP: diastolic blood pressure, BMI: body mass index, CVD: cardiovascular disease.

### Lifetime smoking amount and severity of airflow limitation

**Figure 2:**
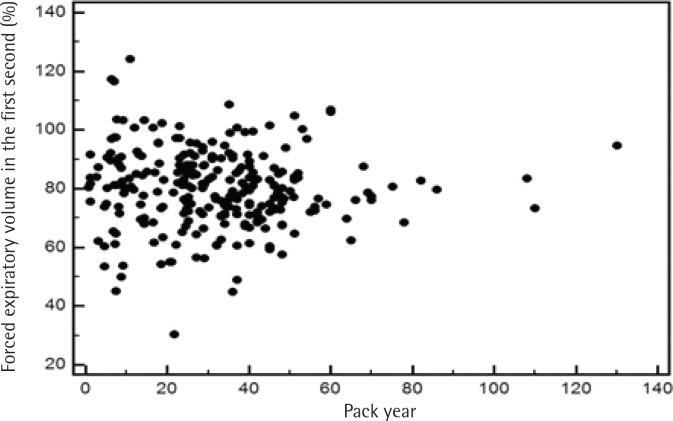
Pearson’s correlation analysis between per cent predicted values of FEV_1_ and total amount of lifetime smoking among current smokers

**Figure 3:**
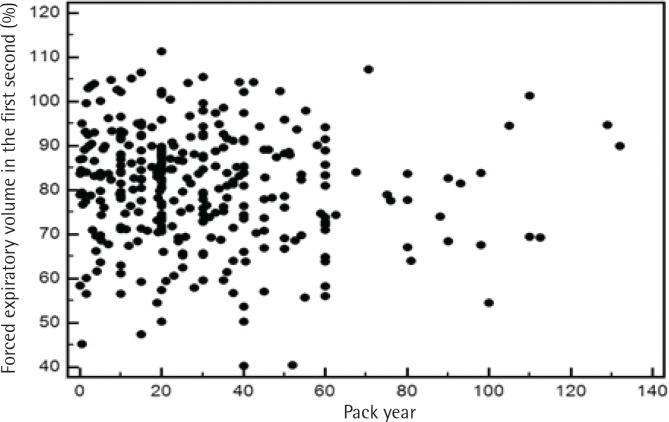
Pearson’s correlation analysis between per cent predicted values of FEV_1_ and total amount of lifetime smoking among former smokers

To address the relationship between severity of airflow limitation and total lifetime smoking amount, a Pearson’s correlation analysis was performed between per cent predicted values of FEV_1_ and total lifetime smoking amount with no significant correlation between the two, among both current and former smokers (Figures 2 and 3).

### Factors associated with the development of airflow limitation among smokers

Table 5 and Supplementary Table 2 summarise the factors associated with the development of airflow limitation. Age, male gender and lifetime smoking amount were significantly associated with the development of airflow limitation after controlling for sociodemographic, nutritional and various health-related conditions. Smokers aged 56–65 years, 66–75 years and ≥76 years were 2.32, 4.33 and 9.74 times, respectively, more likely to have airflow limitation than smokers between 40 and 55 years of age (95% CI: 1.54–3.50; 2.47–7.60; and 3.61–26.27, respectively). Male smokers were 3.03 (95% CI: 1.38–6.68) times more likely to have airflow limitation than female smokers. In terms of lifetime smoking history, an increase of one pack-year corresponded to a 1.02 (95% CI: 1.01–1.03) times higher chance of developing airflow limitation.

**Table 5:** Factors associated with the development of airflow limitation among study participants

*Factors*	*Adjusted OR ( 95% CI)^a^*	*p*
**Age** (ref: 40–55 years)		
56–65	2.32 (1.54–3.50)	<0.001
66–75	4.33 (2.47–7.60)	<0.001
≥76	9.74 (3.61–26.27)	<0.001
**Gender** (ref: Female)		
Male	3.30 (1.38–6.68)	0.006
**Lifetime smoking amount**		
(pack-years)	1.02 (1.01–1.03)	<0.001

Multiple logistic regression analyses, OR: odds ratio, CI: confidential interval.

a Adjusted for age, gender, marital status, employment types, living place, lifetime smoking amount, BMI, dietary intake, comorbidities such as DM, hypertension, CVD, cancer, and heavy drinking, perceived health status, EQ-5D, EuroQoL:VAS, and perceived stress.

## DISCUSSION

The present study investigated the prevalence of airflow limitation, suggestive of having COPD, among smokers, factors associated with airflow limitation among smokers and the relationship between smoking history and the severity of airflow limitation among Korean smokers using the nationally representative cross-sectional survey data publicly available through KNHANES (http://knhanes.cdc.go.kr). Most studies exploring risk factors for COPD have compared smokers and non-smokers, with smoking as only one of the independent variables^26,29^. In this study, we took a different approach, studying only smokers and comparing those with or without airflow limitation. We believe that such a simplified approach would give us a clearer answer regarding why only some smokers develop COPD. Airflow limitation that is only partially reversible with a bronchodilator or is completely irreversible is the characteristic physiological feature of COPD^33,36^. Given the lack of the data of post-bronchodilator FEV_1_ in this study, we adopted the term ‘airflow limitation’ instead of COPD in the present study. The principal findings of this study will be summarised next.

First, the prevalence of airflow limitation among smokers in the present study was approximately 20% (Table 1), which is comparable with rates previously published in cross-sectional studies similar to ours^8^. As mentioned earlier, previous researchers have reported that COPD risk varies according to ethnicity^24,25^. For instance, COPD has a higher prevalence in White than in non-White ethnicities^24,25^. Possible reasons proposed for such ethnic differences include variable pathways for nicotine metabolism or differences in dietary intake of fruit and vegetables, among others^38,39^. Nonetheless, the prevalence of airflow limitation among smokers in the present study was comparable with rates previously observed^40^.

Second, among the many variables we examined, only age, male gender and lifetime smoking amount were significantly associated with the development of airflow limitation (Table 5 and Supplementary Table 2). This indicates that an older Korean man who is a heavy smoker is more likely to develop COPD. The importance of aging in the pathogenesis of COPD has been suggested by others^12^. For example, the incidence of COPD increases with age, with the greatest increase observed in patients aged 65–74 years^11^. Normal aging results in the loss of elastic recoil, stiffening of the chest wall, gas exchange alteration, and decrease in exercise tolerance. These changes are similar to those in patients with emphysema. Thus, aging is considered to be a significant contributor to the development of COPD^12^. Next, a significant association between lifetime smoking amount and airflow limitation was observed, and the prevalence of airflow limitation was significantly higher in male smokers in this study. Although COPD is believed to be more common in men, some researchers suggest that women are actually more susceptible to tobacco-induced lung diseases^13,14^. Because of increased tobacco use among women in high-income countries and the higher risk for exposure to indoor air pollution (such as solid fuel used for cooking and heating) in low-income countries, the disease now appears to affect men and women almost equally^41^.

Third, we examined the relationship between pack-years and the severity of airflow limitation. Although we observed a significant association between pack-years and the prevalence of airflow limitation, oddly, the severity of airflow limitation did not have a significant correlation with pack-years in our study (Figures 2 and 3). According to a recent study, the single best variable for predicting COPD development is a >40 pack-year history of smoking^42^. However, a threshold for daily smoking amount and years of smoking resulting in COPD may vary from one individual to another^9^. Of note, a positive relationship between pack-years and the prevalence of airflow limitation in this study appears not to be due to daily smoking amount but due to years of smoking, as suggested in Table 3. Intriguingly, a recent study has reported that ‘years of smoking’ alone provides risk estimates of COPD^43^. Most studies use pack-years to assess the lifetime smoking amount, but the pack-years value is a measure that assigns the same weight on both daily smoking amount and years of smoking. The Lung Health Study, which included participants from diverse ethnic backgrounds, reported that increasing daily smoking amount is associated with rapidly declining lung function in mild-to-moderate COPD; however, no direct comparisons were made between daily smoking amount and years of smoking^44^. Another cross-sectional study using the COPDGene cohort of current and former smokers with non-Hispanic White or African-American ethnic background showed that ‘years of smoking’ was a more important factor in the development of COPD^43^. Therefore, it would be interesting to identify which factor—daily smoking amount, years of smoking or lifetime smoking amount (pack-years)—is most responsible in the development of COPD according to ethnicity in future studies.

Other findings of our study are also worth mentioning. Although they did not turn out to be significant factors after controlling for multiple variables, most nutritional indices, comorbidities and quality of life were significantly worse in the airflow limitation group when a simple comparison was made (Table 4). Nutritional deficiency in COPD may result from an imbalance between energy intake and consumption. Inadequate intake has been explained by impaired regulation of leptin, a hormone that reduces food intake, in the setting of chronic dyspnoea. The increased energy consumption may be due to the increased work expanded in breathing or increased protein catabolism due to systemic inflammation^45^. Paradoxically, the prevalence of metabolic syndrome is also high in COPD^22^. A recent meta-analysis has shown that healthy diet characterised by high intake of vegetables, fruit, fish and whole-grain products was associated with a decreased risk of COPD, while an unhealthy diet characterised by all kinds of red and processed meats, refined grains, sweets and French fries was associated with an increased risk of COPD^21^. Therefore, it seems that both nutritional deficiency and excess intake, as well as dietary patterns, which could be related to geographical differences, seem to be associated with the development of COPD. It is not clear whether ethnicity plays a different role in the development of COPD according to the nutritional status. Nonetheless, we did not find nutrition a significant factor in the development of airflow limitation among Korean smokers (Tables 4 and 5). COPD is often accompanied by comorbidities, such as cardiovascular disease, metabolic syndrome, DM and gastro-oesophageal reflux^46^. These comorbidities are associated with frequent exacerbation of COPD and increased mortality associated with COPD^47-50^. Although the prevalences of DM, hypertension, CVD and cancers were higher in the group with airflow limitation, we did not find comorbidity as a significant factor in the development of airflow limitation among our study participants (Tables 4 and 5). Also, the level of household income showed no difference between the group without airflow limitation and the group with airflow limitation, suggesting that socioeconomic status might not be a critical factor in the development of airflow limitation among Korean smokers, contrary to what has been previously reported^19^.

In the past, previous researchers have reported that various factors, such as aging^11,12^, gender^13,14^, abnormal lung growth and development^5^, occupational exposure to particles^15^, air pollution^16^, lower socioeconomic status^19,20^, nutrition^21^, obesity^22^ and educational level^23^, were associated with the development of COPD among smokers. In addition, a few studies have examined the factors associated with COPD according to ethnicity or country, and the identified risks were more or less the same as reported herein^17,18,26-29^. However, the effect of air pollution, biomass smoke or chronic respiratory infection such as tuberculosis, often revealed different factors depending on the geographical location^17,18,26-29^. Notably, one study investigated the factors of airflow limitation using the same cohort of KNHANES. Unlike our study, it analysed the prevalence of airflow limitation and the factors associated with airflow limitation in the entire population, including non-smokers. The prevalence of airflow limitation in that study was understandably lower than that reported by us owing to the inclusion of non-smokers in the analysis. The study reported that age, smoking amounts, male gender and having asthma or tuberculosis were associated with higher prevalence of airflow limitation. Further, obesity and higher educational levels were associated with lower prevalence of airflow limitation^23^. Considering that we applied stricter inclusion and exclusion criteria to select the study participants and our data were derived from only smokers, it will be difficult to make a direct comparison between the two studies. However, the other study also reported age, smoking amount and male gender as critical factors associated with the development of COPD among Korean smokers.

### Limitations

Our study has some limitations. To diagnose COPD definitively, post-bronchodilator spirometry data are needed to exclude patients with reversible airflow limitation, such as those with asthma. Unfortunately, the KNHANES data do not include post-bronchodilator spirometry results. However, we believe that most patients with airflow limitation in our study had COPD for the following reasons: we excluded patients with a history of asthma; all those included were smokers; and those with other pulmonary diseases apparent on CXR were excluded. Therefore, we believe that our findings are reliable even without post-bronchodilator spirometry data. However, we recognise that some patients with asthma or asthma COPD overlap (ACO) might have been included. In addition, we could not analyse the effect of medication that the participants were taking as some, such as beta blockers, could influence airflow limitation; furthermore, we could not take into account the effect of exposure to air pollution, occupational hazards and the inhalation of biomass fuel or particles, which are all established factors associated with COPD, due to a lack of detailed information^15,16^.

## CONCLUSIONS

We conducted the present study to identify the factors that make a ‘susceptible smoker’ develop airflow limitation, suggestive of having COPD, among smokers in Korea. We did this using a nationally representative cross-sectional survey by comparing smokers with and without airflow limitation. After controlling for multiple variables, we found that age, male sex and pack-years were the only factors significantly associated with the development of airflow limitation. This study also suggests that among Korean smokers, years of smoking may be a more important factor associated with airflow limitation than daily smoking amount. Our study findings indicate that the best way to prevent the development and progress of COPD is smoking cessation.
