# Supplementary material for: Factors affecting the development of airflow limitation among South Korean smokers
Source: Tob Induc Dis. 2019 Mar 1;17:16. doi: 10.18332/tid/103088 (PMC6751997; doi:10.18332/tid/103088)
Supplement: Supplementary file 1 [file TID-17-16-s1.pdf]

Supplementary Table 1.1. Prevalence of airflow limitation among non-smokers and the study participants (current and former smokers)

Variables	Non-smokers (n = 4,361)		Current smokers (n = 1,217)		Former smokers (n = 1,352)		*p*
	**n**	**% (SE)**	**n**	**% (SE)**	**n**	**% (SE)**	
FEV1/FVC <0.7	260	6.0 (0.51)	261	19.2 (1.34)	322	22.1 (1.41)	<0.001
Normal spirometry	4,101	94.0 (0.51)	956	80.8 (1.34)	1,030	77.9 (1.41)	

P value by chi-square. *Note:* To assess the prevalence of airflow limitation among the overall population who performed spirometry in the survey, the data of non-smokers were also included.

Supplementary Table 1.2. Mean ages of non-smokers and the study participants

Variables	Non-smokers (n = 4,361)		Current smokers (n = 1,217)		Former smokers (n = 1,352)		*p*
	**n**	**Mean age ± SE**	**n**	**Mean age ± SE**	**n**	**Mean age ± SE**	
**FEV1/FVC <0.7**	260	66.28 ± 0.97	261	60.98±0.78	322	64.83±0.82	<0.001
**Normal** spirometry	4,101	54.97±0.27	956	49.13±0.28	1,030	53.45±0.38	<0.001
**Total**	4,361	55.64±0.27	1217	51.40±0.33	1,352	55.97±0.40	<0.001

P values by AVOVA. *Note:* To assess the prevalence of airflow limitation among the overall population who performed spirometry in the survey, the data of non-smokers were also included.

Supplementary Table 2. Factors associated with the development of airflow limitation among Korean smokers

	**Adjusted OR (95% CI)**	***p***
Age (Ref, age 40-55 years)		
56-65	2.32 (1.54 – 3.50)	< 0.001
66-75	4.33 (2.47 – 7.60)	< 0.001
≥ 76	9.74 (3.61 – 26.27)	< 0.001
Gender (Ref, Female)		
Male	3.30 (1.38 – 6.68)	0.006
Marital status (Ref, Married)		
Never-married	0.36 (0.10 – 1.30)	0.118
Others (Divorced, Separate, or Widowed)	1.61 (0.86 - 2.99)	0.137
Employment types (Ref, Clerical work)		
Labor work	0.95 (0.63 – 1.44)	0.809
Unemployed	1.28 (0.81 – 2.02)	0.288
Living Place (Ref, Rural)		
Urban	1.12 (0.77 – 1.63)	0.548
Lifetime smoking amount (pack-years)	1.02 (1.01 – 1.03)	< 0.001
SBP (each 10 mmHg increase)	1.17 (1.00 – 1.38)	0.053
DBP (each 10 mmHg increase)	0.82 (0.63 - 1.07)	0.142
BMI (Kg/ m^2)	0.94 (0.87 -1.00)	0.051
Dietary intake		
Total energy intake (each 100Kcal increase)	1.00 (0.97 -1.02)	0.724
Vitamin A intake (each 100 μgRE increase)	1.00 (0.98 -1.02)	0.977

Vitamin B1 intake (mg)	0.81 (0.62 -1.07)	0.146
Vitamin B2 intake (mg)	1.33 (0.95 -1.86)	0.101
Vitamin C intake (each 10mg increase)	0.98 (0.96 -1.01)	0.110
Total cholesterol (each 10mg/dL increase)	0.99 (0.95 -1.03)	0.538
Diabetes mellitus (Ref, No)		
Yes	1.28 (0.85 – 1.92)	0.240
Hypertension (Ref, No)		
Yes	1.39 (0.93 – 2.09)	0.108
CVD (Ref, No)		
Yes	0.86 (0.39 – 1.09)	0.708
Cancer (Ref, No)		
Yes	0.93 (0.33 – 2.62)	0.883
Heavy Drinker (Ref, No)		
Yes	0.70 (0.48 – 1.03)	0.068
Perceived health status (Very good/Good)		
Fair	0.74 (0.52 – 1.06)	0.1051
Poor/Very poor	1.03 (0.60 – 1.79)	0.906
EQ-5D	0.37 (0.04 – 3.22)	0.368
EuroQoL: VAS	0.99 (0.98 -1.00)	0.207
Perceived stress (Ref, No)		
Yes	0.87 (0.57 – 1.34)	0.533

Multiple logistic regression analyses; Abbreviation: OR: odds ratio; CI: confidential interval; Ref: reference.

© 2019 Kim Y.
